# Supplementary material for: Effect of Medium Chain Triglycerides on the Digestion and Quality Characteristics of Tea Polyphenols-Fortified Cooked Rice
Source: Foods. 2023 Dec 4;12(23):4366. doi: 10.3390/foods12234366 (PMC10706231; doi:10.3390/foods12234366)
Supplement: Supplementary file 1 [file foods-12-04366-s001.zip › foods-2731485-supplementary.pdf]

Supplementary material captions

Table S1. Sensory scoring criteria for tea polyphenols-fortified cooked rice (TP-FCR) with/without MCT.

Figure S1. Sensory quality radar chart of tea polyphenols-fortified cooked rice (TP-FCR) with/without MCT.

Sensory evaluation

The sensory quality of TP-FCR samples was evaluated by 20 laboratory volunteers (10 males and 10 females) aged 20–30 years who were trained to assess quality attributes. Five sensory attributes (color, flavor, hardness, chewiness, and adhesiveness) of TP-FCR were selected for sensory evaluation, with scores ranging from 1 to 10 for each attribute, and the specific evaluation criteria are shown in Table S1. The six TP-FCR samples were coded with random numbers and served in random order to each panelist and each TP-FCR sample was tested blind in triplicate. Radar charts were plotted based on the scores given by the panelists for each attribute of the samples.

The study was conducted in accordance with the Declaration of Helsinki, and the protocol was approved by the Animal Care and Welfare Committee of Jiangxi Agricultural University (JXAULL-2023-11-02). Informed consent was obtained from all subjects involved in the study.

The radar charts of the mean sensory scores of TP-FCR with different addition of MCT are shown in Figure S1, and different MCT levels had a significant effect on the sensory quality of TP-FCR. The color and adhesiveness of TP-FCR with MCT were significantly improved compared with the control TP-FCR. TP-FCR fortified with 3% MCT had the highest sensory scores in hardness and chewiness, indicating that TP-FCR with 3% MCT had the best palatability acceptability. TP-FCR samples fortified with more than 3% MCT had lower sensory scores in hardness and chewiness compared with TP-FCR with 3% MCT, probably due to their harder texture. The results of the sensory evaluation described above were consistent with the instrumental measurements. There

was no significant difference in the flavor sensory scores of TP-FCR, which indicated that MCT did not impart off-flavors to TP-FCR. Overall, the addition of MCT conferred better sensory quality and higher overall acceptability of TP-FCR.

Table S1. Sensory scoring criteria for tea polyphenols-fortified cooked rice (TP-FCR) with/without MCT.

Sensory attributes	Standard of sensory evaluation	score
	Uniform color, shiny	8–10
color	Uneven color, slightly shiny	4–7
	Dull color	1–3
	With rich fragrance	8–10
flavor	With fragrance but weak， no peculiar smell	4–7
	With peculiar smell	1–3
	Moderate hardness	8–10
hardness	Slightly hard or slightly soft	4–7
	Too hard or too soft	1–3
	Moderate chewiness	8–10
chewiness	Slightly weaker chewness	4–7
	Poor chewiness	1-3
	Smooth, not sticky	8–10
adhesiveness	Slightly sticky	4–7
	Sticky	1–3

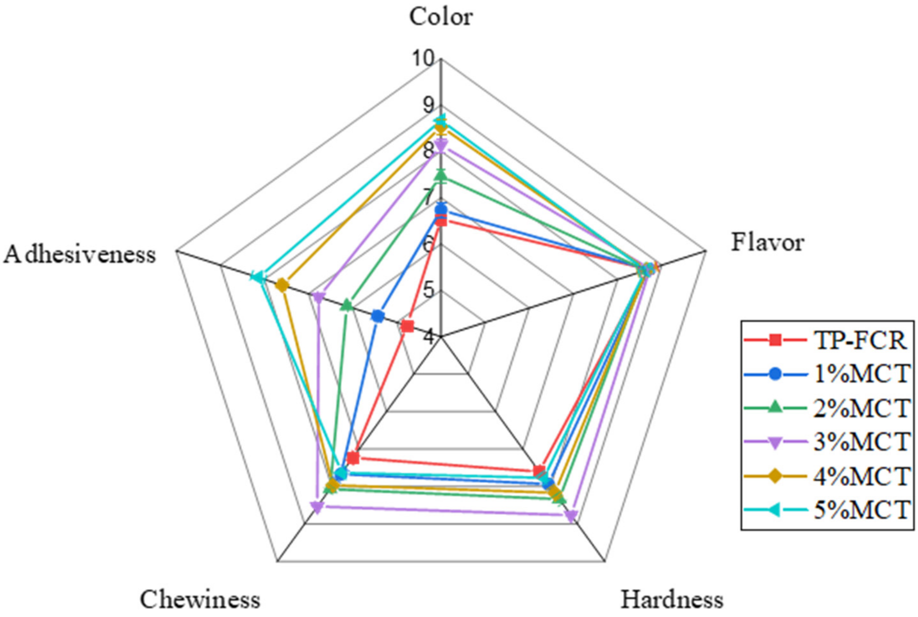
Color
10
9
8
7
6
5
4
Flavor
Adhesiveness
Chewiness
Hardness
TP-FCR
1%MCT
2%MCT
3%MCT
4%MCT
5%MCT

Figure S1.
